# Public objectives and policy instruments for improving the quality of postgraduate education in China

**DOI:** 10.3389/fpsyg.2022.968773

**Published:** 2022-08-24

**Authors:** Erzi Tang

**Affiliations:** School of Economics, Nanjing Audit University, Nanjing, China

**Keywords:** postgraduate education, public policy, educational authority, political perspective, moral education, psychological health

## Abstract

This paper mainly introduces and studies public objectives and instruments in educational policies that authorities can use to improve and evaluate the quality of postgraduate cultivation in China. Under the political environment in the state, the standard for the quality of postgraduate education first includes graduate students in higher education institutions who support the leadership of the Communist Party of China (CPC), educational authorities formulate, and implement policies and regulations surrounding postgraduate education under the leadership of the Party committees as well. From the political perspective, moral cultivation should become an important indicator in the evaluation of the quality of postgraduate education. Specific policy instruments including examination and admission systems, graduation requirements, and performance evaluations are designed to increase knowledge and academic skills or allow students to better perform work in their future careers. Although these policy instruments have played some roles in improving the quality of postgraduate cultivation in practice, some associated social negative phenomena also appear in the field, such as academic misconduct, excessive academic and psychological pressure of postgraduate students, etc. The moral cultivation and psychological health should be measured and assessed while evaluating the quality of postgraduate education. The public objectives surrounding the higher education in political level could provide some useful and constructive recommendations to improve the evaluation system that guides the development of postgraduate education.

## Introduction

Since the 18th National Congress of the Communist Party of China (CPC), the Central Committee of the CPC with Comrade Xi Jinping at the core has paid more attention to Chinese education. In the process of economic growth and social development in China, higher education has played an important role in recent decades. General Secretary Xi Jinping proposes moving faster to transform Chinese universities into world-class universities and develop world-class disciplines and working to maximize the full potential of higher education under the strategy of giving priority to developing education in China at the 19th National Congress of the CPC. To understand how and why policy makers implement some certain policies, and their effects in practice, professional policy analysis could provide a useful method (Browne et al., 2019), e.g., policy decisions contained values, interests, and political contexts decide the direction of educational policies in China. In the speech at a meeting with experts and representatives from education, culture, health and sports sectors (22 September, 2020), Xi Jinping stated that “*We will fully implement the Party’s education policy, give priority to education, cultivate talent for the Party and the country, and develop education that satisfies the demands of the People*.” This important remark has become a guide when policy makers start to formulate higher education policies such as those seeking to improve the quality of postgraduate education. In China, political change had an impact on education, such as basic education and its urban-rural gap (Hannum, 1999). Since the reform and opening-up in China in 1978, a series of educational reforms have been implemented by the Chinese government, and produced some positive effects of educational modernization (Liu and Dunne, 2009). In a word, analyzing how public policies impact educational development, including the issue of improving the quality of postgraduate education, could introduce some useful research questions surrounding political incentives.

In the national education system of China, higher education is a very important part, and plays a significant role in promoting social and economic development, for example, education for sustainable development in higher education in the state provided some innovations surrounding economically effective and environmentally friend development (Niu et al., 2010). From the perspective of economic development/growth, Chen and Feng (2000) indicate that higher education will lead to economic growth, private and semi-private enterprises and international trade in China. However, Brown and Park (2002) find gender bias in education in which academically weak girls have a higher probability of dropping out in primary school and most boys will study in junior secondary school in rural China. Fortunately, the gender gap in education has been decreasing during the last several decades in Chinese rural regions (Dong et al., 2020). Chung and Mason (2012) analyze data and indicate that the quantity of students dropping out of school in poor and rural regions of China may be larger than official statistics admit. These negative phenomena cannot deny the positive influence of educational reforms in China, and Heckman and Li (2004) also suggest that the return to education in China since the early 1990s has increased substantially. Educational return, quality, and equity become the main issues related to the development of education in China (Guo et al., 2019). In education policies, policy makers want to solve the unbalanced structure among different regions such as urban and rural areas in China. The economic development of China simultaneously stimulates the demand for education and increases the ability to pay for educational investment. In the family sector, the cost of children’s education constantly increases and the difference in the emphasis between girls and boys continually decreases since the number of children in a family is limited by China’s family planning policies, e.g., the one-child policy significantly increased the skill development of infants after 6 months (Sylvia et al., 2021). In practice, family background and financial constraints are correlated with higher education attendance in China (Li, 2007). These existing studies provide much useful and constructive evidence to analyze the impact of development and some associated issues on education, including higher education in China.

From a historical perspective, educational activities received great attention from rulers and thinkers in ancient China, especially Confucian scholars and emperors with ruling ideology of Confucianism focused on the development of education. The Confucian tradition of education and approach to teaching thinking still survives from recent classroom research in China (Li and Wegerif, 2014). Confucian educational thought emphasizes the cultivation of personal morality, traditional moral education with Confucian thinking has ongoing value for contemporary moral education (Wang, 2004); therefore, the quality of postgraduate education should include the evaluation of moral education. Some Chinese Confucian classics are often used to guide and study teaching activities and other associated issues, e.g., the nexus between creativity and the doctrine of the mean (Zhongyong) was analyzed based on the traditional Confucian classics (Gao et al., 2022). Regarding the Chinese education system in modern and contemporary times, higher education has constantly and gradually developed since the establishment of the People’s Republic of China. Starting with the reform and opening-up of China, higher education has developed quickly and better serves economic development. Additionally, development of higher education is more reflected in the accumulation of human resources with high quality for the society. Li (2004) summarizes China’s higher education reform from 1998–2003 and indicates many important reviews. According to the Main Statistical Results of National Education Work 2021 in China,^1^ the total number of students in all higher education institutions in China was 44.3 million in the country 2021, and the number of postgraduate entrants was more than 1,176 thousand and the number of postgraduate enrollment was more than 3,332 thousand in the period. Obviously, the scale of higher education in China is very great, and the number of postgraduates is also large all over the world. Hanushek and Wößmann (2007) indicate that cognitive skills rather than mere school attainment are also related to individual earnings and economic growth. Whether highly educated talents can have healthy psychology has become a factor to evaluate the quality of higher education, including postgraduate education, because students undertaking higher education have increased mental health problems (Catling et al., 2022). Postgraduates with high workloads and heavy academic pressure have a higher risk for depression (Zhong et al., 2019). China has a large scale of higher education; thus, the level of higher education quality becomes significant. To evaluate the quality of postgraduate education accurately in the state, the key issue is to reasonably determine the measurement index based on Chinese economic and political environment.

Public sector undertakes major educational tasks in China, especially higher education, including postgraduate education. Government and educational authorities under the leadership of the CPC implement education policies in practice. Studying policy instruments in a new era (since 2012) for improving the quality of postgraduate education and their public objectives is important for researchers and policy-makers to reference some social issues related to higher education. There is a waste of higher education investment in special regions (Bai et al., 2020), and some policies issued designed to change education quality often fail (Cheng and Tam, 1997); nevertheless, many policies can impact the development of higher education in China. When studying the quality of higher education, people often pay attention to the micro level, such as measuring the academic output of some specific universities’ graduates. Additionally, the development of university ranking systems is demanded due to the global expansion of higher education (Dill and Soo, 2005), and the standards of ranking systems also depend on some micro level measures of academic quality in some specific institutions. In short, experts are researching educational questions using student-level evidence; for example, Sánchez et al. (2001) described some failing students at a specific university, *University of Seville*, and their personality characteristics. In a system of higher education, academic achievement is an important evaluation indicator, but Pastor et al. (2015) indicate that the measurement of the research output of higher education institutions (HEIs) is problematic. For similar reasons, analysis at the micro level with academic achievement that describes some factors that impact the quality of postgraduate education may have some difficulties and problems. Hence, this paper mainly reviews some policy instruments for improving the quality of Chinese postgraduate education in macro level. From a political perspective, clarifying the main public objectives and specific policy instruments in many policies surrounding postgraduate education could provide some useful and constructive recommendations to develop higher education in the future.

## General description

In educational activities, the changes of the talents (educated persons) become the main indicator to evaluate the quality of education. General, the quality of postgraduate education mainly contains some indicators as academic skills, knowledge level, working ability, etc. In the Chinese classic Confucian Analects, the Master (Confucius) said, “*In ancient times, men learned with a view to their own improvement. Now-a-days, men learn with a view to the approbation of others*.”^2^ Confucius mainly indicated that people should learn with a vision of their own moral cultivation improvement. The Works of Mencius, as an important Chinese classic, recorded that, “*The great end of learning is nothing else but to seek for the lost mind*.”^3^ This view also showed that the goal of learning in educational activities is to achieve moral improvement. For evaluation of the quality of education, whether moral cultivation is promoted became an important factor in ancient China.

In order to improve the quality of postgraduate education, top designers and policy-makers pay more attention to defining what educational quality means in the development of higher education in contemporary China. Liu et al. (2020) indicate that graduate education should prepare students with global competence to compete globally in the process of globalization. Postgraduates, as candidates for conducting scientific research, obtain more skills to conduct academic work in the future, which could certainly define a higher quality of postgraduate education. However, this definition with only one aspect is not comprehensive, especially in the scenario of China’s educational targets and political environment. Chinese postgraduates, as Chinese people with relatively higher knowledge, must support the leadership of the CPC, which is the most important public objective among China’s educational targets. Government and educational authorities formulate and implement public policies surrounding postgraduate education under the leadership of the CPC. Improving students’ moral level with supporting the party’s leadership becomes the main indicator when evaluating the quality of postgraduate education. For the law-based governance of the country, education managers protect some rights of postgraduates in universities while educating them in complying with disciplines and laws. Many policies were implemented to support postgraduates in completing their studies such as providing national scholarships for students with large amounts of money; the condition of the public sector providing educational subsidies is to maintain and support the leadership of the CPC.^4^ In recent years, some teachers who caused serious harm to postgraduates, such as by molesting female students in universities, have been investigated and punished according to news reports.^5^ If a postgraduate does harm to the country, nation, or other Chinese people, the education manager will also punish the student. In a word, supporting the leadership of the CPC and loving the country are the main public objectives for improving the quality of postgraduate education in China. To achieve this public goal, it is very important to strengthen moral education for graduate students in practice. The Doctrine of the Mean (Zhongyong), as another Chinese Confucian classic, mentioned that, “*He cherishes his old knowledge, and is continually acquiring new*” (see text footnote 3). This learning method is still meaningful for promoting moral education in contemporary China.

**FIGURE 1:**
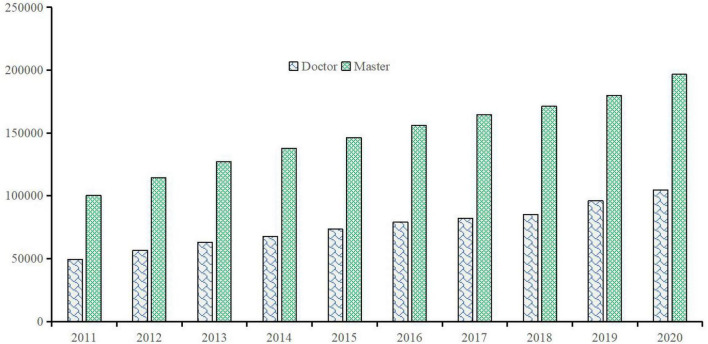
Number of R&D personnel in R&D institutions in China during 2011–2020 (person). Source: The author obtained the data from the China Statistical Yearbook on Science and Technology.

Additionally, a higher quality of postgraduate education is characterized by making greater contributions to realizing the Chinese dream of national rejuvenation from graduates while education managers improve the capacity of education to serve social and economic development. Policy-makers do not think that a postgraduate student only knows how to publish academic papers that could not impact the progress of the economy or society in journals, which is meaningful in China now. To serve the economy and the society, people received graduate education should be more engaged in suitable works. As innovative development has become the main path to achieve sustainable development in an economy, more and more talents are engaged in innovative works becomes the social goal of higher education. For example, Figure 1 portrays the number of R&D personnel, including Doctor and Master, in scientific research and development (R&D) institutions in China during 2011–2020. Over time, the number of personnel with doctoral and master’s degree in R&D institutions gradually increased; therefore, graduate education is of positive significance for serving innovative development in the country.

Clarifying a holistic definition of what the quality of postgraduate education under the Chinese economic and political environment could provide some useful orientations to analyze the policies surrounding educational development in practice. The policies about postgraduate cultivation and management contain these ideas. In the following section, this paper mainly analyzes some specific policy instruments for improving the quality of postgraduate education.

## Postgraduate admission policy

**FIGURE 2:**
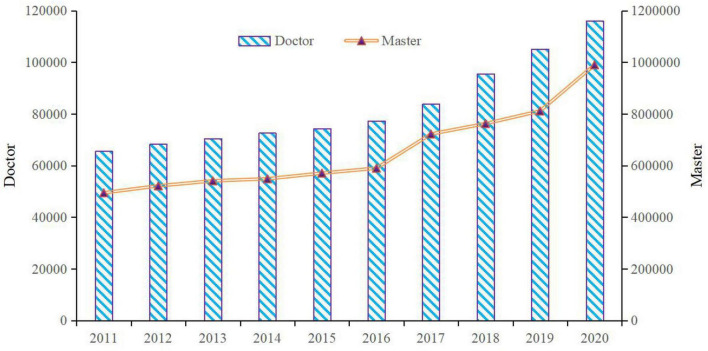
Number of entrants of doctoral and master’s degree in China during 2011–2020 (person). Source: The author obtained the data from the Educational Statistical Yearbook of China. According to the brief introduction in the yearbook, the indicators of entrants of postgraduate students changed since 2017. The changed indicators do not influence analysis in this study.

Due to the scarcity of educational resources, especially the severe shortage of higher education resources, people who want to learn in postgraduate education need to take competitive examinations to obtain a qualification of admission in China. Figure 2 portrays the number of entrants for doctoral and master’s degree in the state during 2011–2020. Although the increasing number of entrants represents the expanding trend of China’s postgraduate admissions, most of the competitors are still unable to study at any university or research institute since the number of candidates for postgraduate examination is much greater than that of the entrants. For example, the number of master’s degree candidates for postgraduate examination in 2020 in China was 3.41 million,^6^ the number in 2019 was 2.9 million,^7^ which is larger than the number of the entrants in the 2 years based on Figure 2, respectively. Due to the fierce competition in this examination, policy-makers want to achieve equity. As the education authority, the Ministry of Education of the PRC formulates the postgraduate admission policy at the national level every year. The master’s degree candidates for postgraduate examinations take national examinations, and the score lines of all disciplines for postgraduate entrance examinations were unified by the education authority. There are 34 universities, such as Peking University, Tsinghua University, Nanjing University, etc., that could solely determine the score line. All other universities and research institutes in China must be in accordance with the score lines that were unified and designated by the official educational authority.^8^

Higher education institutions, including regular HEIs and research institutes, undertake the task of educating postgraduates in China. However, the educational authority and other associated authorities at the national level determine the plan of graduate entrants based on the needs of economic and social development.^9^ These educational institutions shall not enroll more postgraduate students than the plan formulated by the official authorities. Reasonably determining the number of entrants plan becomes an impact factor for the quality of postgraduate education under the Chinese system of educational administration. Although the entrants of postgraduate students gradually increase in China, authorities still strictly control the entrants’ plan comparing to the number of applicants for postgraduate examinations. As a result, passing the examination has always been regarded as a road for success for Chinese people. Although selecting talent through examinations has been severely criticized, the optimal and workable policy using examinations will remain unchanged for China in the future. Postgraduates must have some basic necessary knowledge to successfully complete their study plans. People who pass postgraduate examinations often have relatively more basic knowledge. Hence, a national examination for selecting master’s degree candidates is necessary to improve the current quality of postgraduate education. How to formulate policies to ensure the examinations are open, fair, and just attracts policy-makers’ attention.

The gap between performing academic work well and obtaining high scores on some examinations is relatively large, especially in innovative academic work. China’s education authority divides graduate students into academic and professional graduates. In the master’s degree candidates for postgraduate examination, the examination and the score lines for the two types of graduates are different.^10^ For graduates, doctoral and master’s degree candidates belong to two levels, and the former is higher than the latter. In order to select appropriate students to perform the academic work necessary to become doctoral graduates in many different areas, China’s education authority allows universities and institutes to organize doctoral degree candidates for postgraduate examination themselves. Many Chinese universities have used a method named application—assessment to select doctoral degree candidate postgraduates in recent years, which weakens test scores and pays more attention to scientific research potential. Some appropriate reforms of postgraduate examinations could provide more methods to select the appropriate postgraduate students with academic skills and basic knowledge to educate in practice.

In a word, to ensure that the state selects from talented students and that students, such as postgraduates, grow in a normal way, the guiding principles of examination and admission systems that need to be changed have become the policy direction in China. In practice, some policy changes are playing important roles in improving the quality of postgraduate education, such as application–assessment methods used to select doctoral degree candidates. However, some phenomena surrounding postgraduate examinations should be corrected in the society. Many people give up employment and take postgraduate examination many times, but they could not obtain the qualification of postgraduate admission.^11^ The quality of education contains indicators of serving social and economic development. People who take the postgraduate examinations are basically adults with a bachelor’s degree in China; therefore, their non-participation in work means a huge waste of human resources. Reform of postgraduate admission policy should consider these associated social issues in the future.

## Graduate graduation policy

For most postgraduates, their main aim is to obtain a master’s or doctoral degree in the process of their graduate studies. Although the evaluation of the quality of postgraduate education is a macro-level problem, whether those students in the learning process achieve the public objectives surrounding higher education decides the evaluated results. For education managers and sectors, the quality of postgraduate education for a student is fixed and unchanged when he/she receives their degree and leaves school. Hence, policy-makers should strictly control postgraduates’ graduation to prevent some students with low-level knowledge and skills from receiving a degree. People often criticize Chinese graduate education since a person with a degree such as a master’s or doctoral degree does not have the corresponding ability to do some important work. The policy instrument for controlling postgraduate graduation has become increasingly more obvious in Chinese universities and research institutes in recent years. However, graduation pressure along with academic pressure and depression may cause suicide in Chinese graduate students (Cheng et al., 2020). Policy makers emphasize moral education in the learning process, but educational managers and supervisors of postgraduate programs pay more attention to academic outputs rather than graduate students’ psychological health in the cultivation process. Postgraduates in many Chinese higher educational institutions have to publish one or several papers in academic journals when they want to be awarded a PhD or master degree, especially doctoral degree candidate postgraduates. These requirements for graduate graduation probably cause some academic dishonesty, such as plagiarism (Shen and Hu, 2021). As a result, it is not a good thing that graduate graduation is becoming more and more difficult in higher educational activities.

**FIGURE 3:**
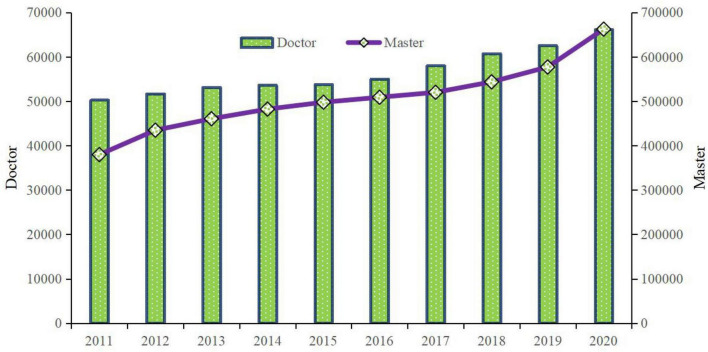
Number of graduates of doctoral and master’s degree in China during 2011–2020 (person). Source: The author obtained the data from the Educational Statistical Yearbook of China.

As described above, education managers divide graduate students into academic and professional graduates to improve the capacity of higher education with graduate cultivation to serve Chinese economic and social development. The two types of graduates’ cultivation processes and graduation requirements are also different; the former emphasizes academic skills, and the latter stresses the importance of serving society and pursuing a career directly. The policy with classified management of postgraduate cultivation is an appropriate method to improve the quality of postgraduate education. Academic graduates with better research skills require more innovation in their theses, and professional graduates with better practical abilities require more references in their theses. Reforms of graduate graduation policy for different types of graduates’ cultivation could also provide some useful recommendations to improve the quality of postgraduate education. Policy that extends the postgraduate education system has become another main method to improve the quality of postgraduate education in China. Especially in the cultivation of doctoral degree postgraduates, most universities and institutes in China have extended the shortest learning time. For example, starting in 2020, Nanjing University established that all doctoral candidates must study at least 4 years before graduation. This policy instrument is often questioned by postgraduates themselves, but obtaining adequate knowledge and skills in the process of postgraduate study requires considerable time. Hence, this policy also has a positive influence on improving postgraduate education quality generally. Additionally, public objectives contain that graduates should be engaged in suitable work. Expanding the scale of postgraduate education indicates that the employment of graduates has become an important social issue. Figure 3 portrays the number of graduates of doctoral and master’s degree in China during 2011–2020. Increasing graduates of doctoral and master’s degree over time represents that appropriately matching talents with jobs becomes an increasingly important indicator in the process of evaluating the quality of postgraduate education in the state.

Educational managers pay more attention to graduate degree theses. People who obtain master’s or doctoral degrees must finish their theses themselves under the guidance of supervisors. Even if a student has obtained the degree and left the school to perform some works, if academic misconduct is discovered with his/her thesis, managers can withdraw the degree.^12^ This is the most serious punishment for people with graduate degrees. This policy instrument with academic supervision and punishment has reduced the incentive for academic misconduct. Lower quality of postgraduate education with more academic misconduct always exists. Hence, this policy instrument is also effective for higher education with higher quality postgraduate cultivation. At no time and under no circumstances should educational authorities forgive academic misconduct. To graduate, postgraduate students should study hard to improve academic skills and enrich scientific knowledge. Policies surrounding graduate graduation demand some corresponding policies support; therefore, educational authorities should increase the academic subsidies of postgraduate students. Especially, whether some graduate graduation policies are implemented effectively depends on postgraduate admission policy, because selecting suitable candidates could help postgraduate students graduate on time. In a word, the two policies have a nexus in higher educational activities under the environment with Chinese economic and political characteristics.

## Performance evaluation policy

**FIGURE 4:**
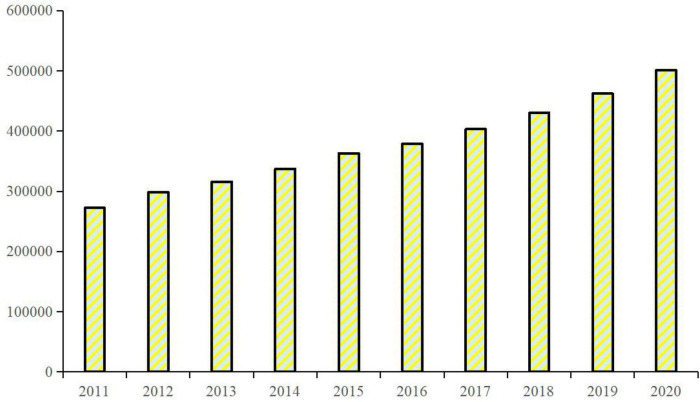
Number of supervisors of postgraduate programs in China during 2011–2020 (person). Source: The author obtained the data from the Educational Statistical Yearbook of China.

In China, nation, society, educational institutions, family, and individual input many resources to implement educational activities, respectively. However, their objectives are different, for example, students and their parents mainly want to obtain high score to enter a good school and find a good job based on master’s or doctoral degree, which is inconsistent with the public goal of national education. Hence, how to evaluate the performance of educational activities becomes a difficult social issue in practice. In the speech at the National Education Conference (10 September, 2018), Xi Jinping stated that, “*We should get rid of this obsession with scores, enrollment rates, diplomas, academic papers and professional titles, remove their excessive influence on the evaluation of the education system, and reverse the utilitarian trend in education*.” The utilitarian trend in higher education is shown as researchers, including postgraduate students and their supervisors, mainly focus on the published papers and fund projects, etc. In the postgraduate cultivation process, students are directly connected with their supervisors, and educational authorities and universities or research institutes formulate some associated policies to manage postgraduate education. Hence, teachers or researchers who are supervisors become the first people who are responsible for the quality of postgraduate education. Figure 4 portrays the number of supervisors of postgraduate programs in China during 2011–2020 and shows that increasing supervisors could provide educational support suitable for the expansion of postgraduate education in the state. The evaluation of supervisors depends on the achievements of his/her postgraduates. Educational authorities increasing graduation requirements for postgraduates most often allows for the best selection of supervisors at Chinese universities and institutes. A bad relationship between supervisors and postgraduates is often reported, and some students commit suicide to end their studies.^13^ Selecting appropriate supervisors becomes an indicator for the evaluation of the quality of postgraduate education in China.

For universities and research institutes, qualifying to enroll graduate students means they could obtain more financial funds for education in China. However, this qualification dynamically changes and is impacted by the quality of postgraduate education, which is evaluated by educational authorities. The Academic Degrees Committee of the State Council (Ministry of Education) releases dynamic adjustments to the withdrawn and added lists of degree authorization points every year.^14^ In order to prevent some degrees from being issued, authorization points will be withdrawn by authorities, and policy-makers at universities and institutes seek to increase the graduation requirements for postgraduates, which are beneficial to ensuring the postgraduate cultivation quality in general. The extension of the postgraduate education system and severe punishment for academic misconduct described above are other policy instruments from managers. In the Chinese classic Confucian Analects, Tsze-hea stated, “*The officer, having discharged all his duties, should devote his leisure to learning. The student, having completed his learning, should apply himself to be an officer*” (see text footnote 2). Universities and institutes have gradually reduced the quantity of graduate students with targeted employment who have mainly come from officers in recent years, because they may not have enough time to finish their postgraduate studies. In short, as a postgraduate training unit, universities and research institutes with evaluation pressure from education authorities have formulated many new policy instruments to improve postgraduate cultivation quality in the state. Evaluation pressure for postgraduate students, supervisors, universities and research institutes could provide some motivation in the process of graduate education, but also expand the utilitarian trend. Some policy instruments surrounding the direct evaluation of the quality of postgraduate education should also be supported by some associated policies in practice, especially the policy of moral education and mental health education in the process of postgraduate cultivation.

The education authorities at the provincial level, such as the Jiangsu Education Department, simultaneously accept the leadership of the People’s Government of Jiangsu Province and the Ministry of Education of the PRC. These administrative bodies implement public policies under the leadership of Party committees in China. There is a promotion tournament of local officials in China; thus, the local government hopes that the education quality is relatively higher in its own region, which is beneficial to the assessment of officers. The education authority at the provincial level will formulate some detailed rules and regulations under the guidance of the Ministry of Education of the PRC to improve the postgraduate cultivation quality in its own region. For example, the Jiangsu Education Department evaluates some master’s degree theses from authors that have graduated *via* strictly random sampling from all universities and institutes located in the region. The results of the evaluation with some institutions having low-quality theses are publicly announced on the website.^15^ This method becomes a very strict way of conducting performance evaluations and causes universities and research institutes to pay more attention to improving postgraduate education quality in practice. For the development of science and technology, acquiring innovative knowledge becomes the most important way (Tang et al., 2014), many articles published in academic journals contribute little to none to the scholarly body of knowledge and may be categorized as “scholarly bullshit” (Kirchherr, 2022). The evaluation pressure of postgraduate education should be transformed into an innovation incentive under the vision of innovative development in China. In a word, the orientation of educational evaluation should reflect the public objectives in the state; therefore, educational authorities should use some appropriate policy instruments to assess the quality of postgraduate education. The degree of evaluation pressure should be moderate, and the indicator of educational evaluation should contain some measurement of moral cultivation and psychological health in the future.

## Conclusion

In the process of building socialism with Chinese characteristics for a new era, the CPC exercises overall leadership over all areas and endeavors in every part of the country, which has become the main feature of the political environment in the state. Educational authorities formulate and implement public policies under the leadership of the CPC in practice; therefore, the public objectives of educational policies inevitably contain political nature. The main purpose of education is to cultivate talents, especially higher education talents, who support the leadership of the CPC. Hence, policy-makers evaluate the quality of postgraduate education in China by assessing whether graduate students support leadership. The Chinese education authorities formulate policies to ensure that graduate students behave and perform in accordance with the interests of the CPC and the people, such as complying with discipline and laws. Regarding detailed policies, education authorities at the national and provincial levels have also implemented policies to improve the quality of postgraduate cultivation by increasing knowledge and academic skills and performing better work that society needs. Some policy instruments for examination and admission systems, graduation requirements, and performance evaluations of postgraduate education have positive effects on improving postgraduate education quality in practice.

The political nature and public objectives in educational policies indicate that moral education and its evaluation should be contained in the quality of postgraduate education in China. To serve social and economic development, talents educated in postgraduate education should be engaged in suitable work. Their psychological health and moral cultivation should also become a component of the quality of postgraduate education. These detailed educational policies surrounding postgraduate admission, graduation, and performance evaluation, need to reform under the mechanism to promote education on values and moral integrity. Give prominence to political perspectives along with the introduction of the quality of postgraduate education could provide some useful and constructive recommendations to reform educational policies and correct the warped evaluation system in China, because educational authorities in the state implement policies and regulations that guide the development of higher education under the leadership of the CPC.

## Author contributions

ET made all work in this study and approved the submitted version.

## Footnotes

1 For details, see http://www.moe.gov.cn/jyb_xwfb/gzdt_gzdt/s5987/202203/t20220301_603262.html.

2 This is English version translated by James Legge (1815–1897) who was a Scottish sinologist, and published by Liaoning People’s Publishing House, 2016.

3 This is English version translated by James Legge, and published by Liaoning People’s Publishing House, 2017.

4 For details, see: http://www.moe.gov.cn/jyb_xxgk/moe_1777/moe_1779/201210/t20121022_143547.html.

5 One case can be seen at https://www.sdjzu.edu.cn/jwjcs/info/1030/3426.htm.

6 For details, see: https://yz.chsi.com.cn/kyzx/kydt/201912/20191218/1860475466.html.

7 For details, see: https://yz.chsi.com.cn/yzzt/fxbg2019.

8 For details surrounding 2022 examination, see: https://yz.chsi.com.cn/kyzx/zt/kyfs2022.shtml.

9 The policy in China 2022, see: https://yz.chsi.com.cn/kyzx/jybzc/202109/20210903/2105941509.html.

10 https://yz.chsi.com.cn/kyzx/kydt/202103/20210312/2037434816.html provides the different score lines in 2021 and https://yz.chsi.com.cn/kyzx/kydt/202203/20220311/2172338980.html provides them in 2022.

11 One case could be seen at: https://view.inews.qq.com/a/20220712A06NM200?refer=wx_hot.

12 Two cases could be seen at: https://www.thecover.cn/news/9510669.

13 For one case, see: https://new.qq.com/omn/20200210/20200210A0SXY600.html?pc.

14 The website, http://www.moe.gov.cn/srcsite/A22/yjss_xwgl/moe_818/202111/t20211112_579362.html, provides the information for 2020.

15 For the 2019 result, see: http://jyt.jiangsu.gov.cn/art/2020/3/13/art_58320_9010742.html.
